# Correction: Non-pharmaceutical interventions for people living with HIV with cognitive impairment: A scoping review

**DOI:** 10.1371/journal.pone.0330546

**Published:** 2025-08-19

**Authors:** Lucinda Stuart, Kate Alford, Jaime H. Vera

The third author’s name is spelled incorrectly. The correct name is: Jaime H. Vera. The correct citation is: Stuart L, Alford K, Vera JH (2024) Non-pharmaceutical interventions for people living with HIV with cognitive impairment: A scoping review. PLoS ONE 19(11): e0314185. https://doi.org/10.1371/journal.pone.0314185.
