# Supplementary material for: Non-pharmaceutical interventions for people living with HIV with cognitive impairment: A scoping review
Source: PLoS One. 2024 Nov 25;19(11):e0314185. doi: 10.1371/journal.pone.0314185 (PMC11588236; doi:10.1371/journal.pone.0314185)
Supplement: S1 Table — (DOCX) [file pone.0314185.s002.docx]

**S2 Table. Demographic data from studies included in this review, used to calculate mean and standard deviation for age, and interquartile range for intervention length**

| **Study** | **N (randomised)** | **N (completed)** | **N (Male) for those completed** |  |  | **Age (Mean)** | **Age (SD)** |  |  | **Intervention Length (weeks)** |
| --- | --- | --- | --- | --- | --- | --- | --- | --- | --- | --- |
| ***Byun et al*** | ***41*** | ***41*** | ***29*** |  |  | **54.6** | **6.6** |  |  | **12** |
| ***Ceccarelli et al*** | ***35*** | ***35*** | ***33*** |  |  | **46.7** | **12.4** |  |  | **24** |
| ***Etesami et al*** | ***60*** | ***49*** | ***32*** |  |  | **37.8** | **7.6** |  |  | **12** |
| ***Frain et al*** | ***24*** | ***22*** | ***19*** |  |  | **56.0** | **5.2** |  |  | **8** |
| ***Henry et al*** | ***21*** | ***21*** | ***18*** |  |  | **51.8** | **2.6** |  |  | **16** |
| ***Hossain et al*** | ***3*** | ***3*** | ***2*** |  |  | **54.7** | **3.6** |  |  | **15** |
| ***Mayo et al*** | ***53*** | ***53*** | ***49*** |  |  | **58.1** | **7.0** |  |  | **9** |
| ***Morrison et al*** | ***14*** | ***14*** | ***6*** |  |  | **56.7** | **5.7** |  |  | **12** |
| ***Nweke et al*** | ***73*** | ***73*** | ***not known*** |  |  |  |  |  |  | **12** |
| ***Ownby et al*** | ***14*** | ***11*** | ***11*** |  |  | **51.5** | **4.7** |  |  | **2** |
| ***Ownby et al*** | ***46*** | ***44*** | ***37*** |  |  | **57.1** | **5.0** |  |  | **2** |
| ***Towe et al*** | ***21*** | ***21*** | ***16*** |  |  | **47.9** | **11.2** |  |  | **10** |
| ***Vance et al (2021)*** | ***88*** | ***88*** | ***60*** |  |  | **54.2** | **7.0** |  |  | **12** |
| ***Vance et al***  ***(2023)*** | ***216*** | ***114*** | ***71*** |  |  | **51.5** | **6.3** |  |  | **15** |
|  |  |  |  |  |  |  |  |  |  |  |
|  | **709** | **589** | **383** |  | **Total** | **52.2** | **5.53** |  | **Median** | **12** |
|  |  |  |  |  |  |  |  |  | **Q1** | **8.5** |
|  |  |  |  |  |  |  |  |  | **Q3** | **15** |
|  |  |  |  |  |  |  |  |  | **IQR** | **6.5** |
