# Supplementary material for: Non-pharmaceutical interventions for people living with HIV with cognitive impairment: A scoping review
Source: PLoS One. 2024 Nov 25;19(11):e0314185. doi: 10.1371/journal.pone.0314185 (PMC11588236; doi:10.1371/journal.pone.0314185)
Supplement: Attachment — Submitted filename: Response to Reviewers.docx [file pone.0314185.s003.docx]

**Response to Reviewers regarding “Non-pharmaceutical interventions for people living with HIV with cognitive impairment: a scoping review”**

Dear Reviewers,

I would like to thank the reviewers for providing thoughtful and constructive feedback on my manuscript. I have carefully considered all the comments, and I have made the necessary revisions in response to their comments. Below, I have provided a detailed response to each of the reviewers' remarks. I hope that the revised manuscript meets the expectations of both the reviewers and the academic editor.

| **Reviewer Comment** | **Response/Action** |
| --- | --- |
| Journal Requirements | |
| 1. Please ensure that your manuscript meets PLOS ONE's style requirements, including those for file naming. | Correction made. |
| 2. Please note that funding information should not appear in any section or other areas of your manuscript. We will only publish funding information present in the Funding Statement section of the online submission form. Please remove any funding-related text from the manuscript. | Text removed. |
| 3. Please provide an amended Competing Interests Statement that explicitly states this commercial funder, along with any other relevant declarations relating to employment, consultancy, patents, products in development, marketed products, etc. Within this Competing Interests Statement, please confirm that this does not alter your adherence to all PLOS ONE policies on sharing data and materials by including the following statement: "This does not alter our adherence to PLOS ONE policies on sharing data and materials.” If there are restrictions on sharing of data and/or materials, please state these. Please note that we cannot proceed with consideration of your article until this information has been declared. Please include your amended Competing Interests Statement within your cover letter. We will change the online submission form on your behalf. | An amended Competing Interests Statement has been included with the cover letter, and described here: *JHV has received honoraria and research grants from and been a consultant or investigator in trials sponsored by Merck, Janssen Cilag, Piramal and Gilead Sciences. He has received sponsorship to attend scientific conferences from Janssen Cilag, Gilead Sciences and AbbVie. This does not alter our adherence to PLOS ONE policies on sharing data and materials. Funding for presentations and investigator sponsor grants but not related to this work.* |
| 4. Please confirm at this time whether or not your submission contains all raw data required to replicate the results of your study. Authors must share the “minimal data set” for their submission. PLOS defines the minimal data set to consist of the data required to replicate all study findings reported in the article, as well as related metadata and methods (<https://journals.plos.org/plosone/s/data-availability#loc-minimal-data-set-definition>). For example, authors should submit the following data: - The values behind the means, standard deviations and other measures reported; - The values used to build graphs; - The points extracted from images for analysis. Authors do not need to submit their entire data set if only a portion of the data was used in the reported study. If your submission does not contain these data, please either upload them as Supporting Information files or deposit them to a stable, public repository and provide us with the relevant URLs, DOIs, or accession numbers. For a list of recommended repositories, please see <https://journals.plos.org/plosone/s/recommended-repositories>. If there are ethical or legal restrictions on sharing a de-identified data set, please explain them in detail (e.g., data contain potentially sensitive information, data are owned by a third-party organization, etc.) and who has imposed them (e.g., an ethics committee). Please also provide contact information for a data access committee, ethics committee, or other institutional body to which data requests may be sent. If data are owned by a third party, please indicate how others may request data access. | Raw data submitted as a Supplementary Information file (S2 Table). |
| 5. [Please include captions for your Supporting Information files at the end of your manuscript, and update any in-text citations to match accordingly.](http://c05y1x9s.r.us-east-2.awstrack.me/L0/http:%2F%2Fjournals.plos.org%2Fplosone%2Fs%2Fsupporting-information./1/010f01928a991d7a-d53f2f61-1168-46fc-8f7e-73a9715d75cf-000000/T9ZmKSgdvdvuZjc1Q834Aa6QtrQ=180) | Correction made. |
| 6. Please review your reference list to ensure that it is complete and correct. If you have cited papers that have been retracted, please include the rationale for doing so in the manuscript text, or remove these references and replace them with relevant current references. Any changes to the reference list should be mentioned in the rebuttal letter that accompanies your revised manuscript. If you need to cite a retracted article, indicate the article’s retracted status in the References list and also include a citation and full reference for the retraction notice. | Reference list is complete and correct. |
| Reviewer 1 | |
| 7. Page 6, line 123 – remove comma after Despite. | Correction made. |
| 8. Page 7, lines 138 – Is this acceptability of interventions? | Correction made. |
| 9. Page 9, lines 188 – When starting a sentence with a number, it should be spelled out. | Correction made. |
| 10. Table with Byun intervention details – It was speed of processing and attention as standard, that is if there were deficits in those domains, otherwise other cognitive domains with corresponding training was conducted. That is the way for the TOPS study as well. | Thank you for the clarification. I have added more detail within Table 1, and within the text (lines 265-268) to emphasise this point more clearly. |
| 11. Page 23, line 286 – remove comma after sessions. | Correction made. |
| 12. Page 23 – There are some HIV transcranial studies missing from this search. o Fazeli, P. L., Woods, A. J., Pope, C. N., Vance, D. E., & Ball, K. K. (2019). The effect of transcranial direct current stimulation combined with cognitive training on cognitive functioning in older adults with HIV: A pilot study. Applied Neuropsychology: Adult, 26(1), 36-47. o Pope, C. N., Stavrinos, D., Vance, D. E., Woods, A. J., Bell, T. R., Ball, K. K., & Fazeli, P. L. (2018). A pilot investigation on the effects of combination transcranial direct current stimulation and speed of processing cognitive remediation therapy on simulated driving behavior in older adults with HIV. Transportation Research Part F: Psychology and Behavior, 58, 1061-1073. o Cody, S. L., Fazeli, P. L., Crowe, M. G., Kempf, M-C., Moneyham, L., Stavrinos, D., & Vance, D. E. (2020). The effects of speed of processing training and transcranial direct current stimulation on global sleep quality and speed of processing in older adults with and without HIV: A pilot study. Applied Neuropsychology: Adult, 27(3), 267-278. | Thank you for your suggestions. Fazeli et al. was excluded as it was unclear which participants had cognitive impairment, and the degree of this cognitive impairment. Those with and without cognitive impairment were not separated for reporting results and analysis. Pope et. al and Cody et al. were excluded as participants baseline cognitive function was not assessed, and our study was looking at the neurocognitive outcomes in those with pre-existing cognitive impairment. |
| Reviewer 2 | |
| 13. *‘This indicates a role for non-pharmaceutical interventions, which aside from potentially improving neuropsychological outcomes may be effective at improving self-reported functional abilities and QoL’. and ‘It is of interest, that for most interventions included in this review, the main outcome was improvement in neuropsychological test scores. Recent community-based approaches have documented a shift in patient priorities and calls to develop local and feasible interventions which improve HRQL and daily life for those with HIV and cognitive difficulties’.*   Could the authors comment on this discrepancy and how the patient’s perspective and priorities could be better integrated in the design of non-pharmacological studies. Should studies focus on people with impairment on neuropsychological testing (and how would this eventually be transferred to the clinic setting as such testing is usually not available outside the research setting?), or on people with cognitive complaints? And what should the outcomes be? Currently, there is a lack of clarity about what the explanatory framework and how it influences study design. Is the hypothesis that improving performance on a cognitive test will improve function and quality of life, as is explicitly stated in the referenced paper regarding the ACTIVE study? Comment on this issue would enrich the paper. | Thank you for your point, I have tried to elaborate and discuss the issues raised in (line 442-453) and (line 460-472). |
| 14. *‘Establishing a standardised cognitive battery for use in experimental studies investigating CI in people living with HIV would be extremely beneficial to future work, to facilitate comparison between studies and help identify which interventions are the most valuable.’*   This statement seems in contradiction with the point regarding patient’s’ priorities. Please elaborate. | Addressed above. |
| 15. When reporting studies of cognitive training, it would be important to specify if the intervention was delivered in person or performed independently, and the documented adherence, especially if administered at home. While unsupervised administration is less costly, adherence to the intervention is an important (and problematic) aspect. | Thank you for suggesting this data should be included. I have now included this within Table 1. |
| 16. Approximately 14% of people living with HIV experience objective CI: the study quoted was conducted among adults >50 years, a group more at risk: please mention the age group. | Thank you for your point. The age group has now been mentioned (lines 66-67). |
| 17. ‘The Training on Purpose Study (TOPS) examined 20 hours of individualized CCT over 262 12 weeks, targeting specific cognitive deficits identified at baseline. While the study did not reverse a HAND diagnosis for most participants, it did improve global clinical 264 ratings’. (ref missing) | Correction made. |
| 18. ‘In contrast to the findings of TOPS, this study found lower global clinical rating scale scores in the control group both immediately and one year post-intervention than in both arms’: This statement is not clear. Lower than what? The original paper states: Contrary to expectations, those in the control group had slight improvements in global function. | Thank you for this point. Clarification of the findings has been made in lines 282-284. |
| 19. The iSTEP intervention: there is no mention of the findings on cognition. Please add. | Thank you for your point, findings related to cognition for the iSTEP intervention have now been included in lines 360-361 |
| 20. A couple of typos:   Despite, some HIV guidelines: no comma Simulate further consideration: stimulate | Corrections made. |

Thank you for your consideration, and I hope that the above changes to the manuscript are satisfactory.

Yours faithfully,

Lucinda Stuart

5th Year Medical Student
